# Applying science to pressing conservation needs for penguins

**DOI:** 10.1111/cobi.13378

**Published:** 2019-08-13

**Authors:** P.D. Boersma, P. García Borboroglu, N.J. Gownaris, C.A. Bost, A. Chiaradia, S. Ellis, T. Schneider, P.J. Seddon, A. Simeone, P.N. Trathan, L.J. Waller, B. Wienecke

**Affiliations:** ^1^ Center for Ecosystem Sentinels and Department of Biology University of Washington Seattle WA 98103 U.S.A.; ^2^ Global Penguin Society Puerto Madryn 9120 Argentina; ^3^ CESIMAR CCT Cenpat‐CONICET 9120 Puerto Madryn Chubut Argentina; ^4^ Centre d'Etudes Biologiques de Chizé 79360 Villiers‐en‐Bois France; ^5^ Conservation Department Phillip Island Nature Parks Cowes VIC 3922 Australia; ^6^ International Rhino Foundation Strasburg VA 22657 U.S.A.; ^7^ Detroit Zoological Society Royal Oak MI 48067 U.S.A.; ^8^ Department of Zoology University of Otago Dunedin 9016 New Zealand; ^9^ Facultad de Ciencias de la Vida Universidad Andres Bello Santiago 8370146 Chile; ^10^ British Antarctic Survey Cambridge CB3 0ET U.K.; ^11^ Southern African Foundation for the Conservation of Coastal Birds (SANCCOB) Cape Town 7441 South Africa; ^12^ Department of Biodiversity and Conservation Biology University of the Western Cape Bellville Cape Town 7535 South Africa; ^13^ Australian Antarctic Division Kingston TAS 7050 Australia

**Keywords:** climate change, ecosystem sentinels, knowledge gaps, marine spatial planning, nonbreeding habitat, pairwise ranking, science communication, cambio climático, centinelas de ecosistemas, clasificación por pares, comunicación científica, hábitat no reproductor, planificación marina espacial, vacíos de conocimiento

## Abstract

More than half of the world's 18 penguin species are declining. We, the Steering Committee of the International Union for Conservation of Nature Species Survival Commission Penguin Specialist Group, determined that the penguin species in most critical need of conservation action are African penguin *(Spheniscus demersus)*, Galápagos penguin *(Spheniscus mendiculus)*, and Yellow‐eyed penguin *(Megadyptes antipodes)*. Due to small or rapidly declining populations, these species require immediate scientific collaboration and policy intervention. We also used a pairwise‐ranking approach to prioritize research and conservation needs for all penguins. Among the 12 cross‐taxa research areas we identified, we ranked quantifying population trends, estimating demographic rates, forecasting environmental patterns of change, and improving the knowledge of fisheries interactions as the highest priorities. The highest ranked conservation needs were to enhance marine spatial planning, improve stakeholder engagement, and develop disaster‐management and species‐specific action plans. We concurred that, to improve the translation of science into effective conservation for penguins, the scientific community and funding bodies must recognize the importance of and support long‐term research; research on and conservation of penguins must expand its focus to include the nonbreeding season and juvenile stage; marine reserves must be designed at ecologically appropriate spatial and temporal scales; and communication between scientists and decision makers must be improved with the help of individual scientists and interdisciplinary working groups.

## Abstract 2

*Article impact statement*: Safeguarding the future of penguins requires collaboration among scientists and policymakers and immediate, informed conservation action.

## Abstract (es)

Aplicación de Ciencia en las Necesidades de Conservación Urgentes para los Pingüinos.

**Resumen**

Más de la mitad de las 18 especies de pingüinos del mundo están disminuyendo. Nosotros, el Comité Directivo de la Unión Internacional para la Conservación de la Naturaleza, Grupo de Especialistas en Pingüinos, determinamos que las especies de pingüinos con necesidades críticas de conservación son el pingüino africano (*Spheniscus demersus*), el pingüino de las Galápagos (*Spheniscus mendiculus*) y el pingüino de ojos amarillos (*Megadyptes antipodes*). Debido a que sus poblaciones son pequeñas o están declinando rápidamente, estos pingüinos requieren colaboración científica e intervención política inmediatas. También utilizamos un método de clasificación por pares para priorizar las necesidades de investigación y conservación para todas las especies de pingüinos. Entre las 12 áreas de investigación que identificamos, las más prioritarias fueron: cuantificación de las tendencias poblacionales, estimación de las tasas demográficas, predicción de las patrones de cambio ambiental y mejora del conocimiento de las interacciones con pesquerías. Las mayores necesidades de conservación fueron: optimizar la planificación marina espacial, mejorar la colaboración de las partes interesadas y desarrollar planes de manejo de desastres y de acción para cada especie. Coincidimos en que, para mejorar la traducción de la ciencia en la conservación efectiva de los pingüinos, la comunidad científica y los organismos financiadores deben reconocer la importancia de la investigación a largo plazo y apoyarla; la investigación sobre pingüinos y su conservación debe expandir su enfoque para incluir la época no reproductiva y la etapa juvenil; las reservas marinas deben ser diseñadas a escalas espaciotemporales ecológicamente apropiadas; y la comunicación entre científicos y tomadores de decisiones debe mejorar con la ayuda de científicos individuales y grupos de trabajo interdisciplinario.

## Introduction

Penguins are in trouble. Ten of the 18 recognized penguin species are threatened (IUCN 2018) (Table 1), making them the most threatened group of seabirds after albatrosses and petrels (Croxall et al. 2012). More than half of the 18 species are in decline, and species with stable or increasing global populations are sometimes in decline regionally (e.g., Magellanic penguins [*Spheniscus magellanicus*]) (Pozzi et al. 2015). For some species, data are insufficient to estimate global population size.

**Table 1:** Basic information on the 18 species of penguins

Species^a^	IUCN status 2012, b	IUCN status 2018, b	Population trend (IUCN 2018)	Main breeding colonies and foraging range	Maximum recorded nonbreeding range (one‐way)
Emperor (*Aptenodytes forsteri*)	NT	NT	unknown	polar	approximately 7000 km (juveniles) (Wienecke et al. 2010) >1000 km (adults) (Kooyman et al. 2004)
King (*Aptenodytes patagonicus*)	LC	LC	increasing	subantarctic	2650 km (F. Orgeret & C.A. Bost, data)
Adélie (*Pygoscelis adeliae*)	NT	LC	increasing	polar	approximately 4000 km (juveniles), >2700 km (adults) (Clarke et al. 2003)
Chinstrap (*Pygoscelis antarctica*)	LC	LC	decreasing	subantarctic	4000 km (Hinke et al. 2015)
Gentoo (*Pygoscelis papua*)	NT	LC	stable	subantarctic	unknown
Macaroni (*Eudyptes chrysolophus*)	VU	VU	decreasing	subantarctic	approximately 3000 km (Bost et al. 2009)
Royal (*Eudyptes schlegeli*)	VU	NT	stable	subantarctic	unknown
Northern rockhopper (*Eudyptes moseleyi*)	EN	EN	decreasing	subantarctic	> 2000 (Thiebot et al. 2012)
Southern rockhopper (*Eudyptes chrysocome*)	VU	VU	decreasing	subantarctic	approximately 2500 km (Thiebot et al. 2012)
Fiordland (*Eudyptes pachyrhynchus*)	VU	VU	decreasing	Oceania	approximately 2500 km (Mattern et al. 2018)
Snares (*Eudyptes robustus*)	VU	VU	stable	Oceania	unknown
Erect‐crested (*Eudyptes sclateri*)	EN	EN	decreasing	Oceania	unknown
African* (*Spheniscus demersus*)	EN	EN	decreasing	Africa	up to approximately 600 km (juveniles) (Sherley et al. 2017) up to approximately 4000 km (pre‐ and postmoulters) (Harding 2013; Roberts 2016)
Galápagos* (*Spheniscus mendiculus*)	EN	EN	decreasing	South America (equatorial)	approximately 150 km (P.D. Boersma, data)
Humboldt (*Spheniscus humboldti*)	VU	VU	decreasing	South America (SE Pacific)	approximately 1000 km (postbreeding adults) (Pütz et al. 2016)
Magellanic (*Spheniscus magellanicus*)	NT	NT	stable/decreasing	South America	approximately 4000 km (Stokes et al. 2014)
Little (*Eudyptula minor*)	LC	LC	stable	Oceania	approximately 1000 km
Yellow‐eyed* (*Megadyptes antipodes*)	EN	EN	decreasing	Oceania (New Zealand)	approximately 150 km (M. Young, data)

a Species with an asterisk are those ranked as being of the most immediate conservation concern based on a vote by the Steering Committee of the International Union for Conservation of Nature Species Survival Commission Penguin Specialist Group.

b International Union for Conservation of Nature conservation status: LC, least concern; VU, vulnerable; NT, near threatened; EN, endangered.

The International Union for Conservation of Nature Species Survival Commission (IUCN SSC) Specialist Groups consist of members who provide the highest level of scientific rigor and expertise regarding the conservation of the species within their purview (IUCN 2017). We, the IUCN SSC Penguin Specialist Group (PSG) Steering Committee (Supporting Information), held a 2‐day workshop to develop a consensus on the penguin species of most immediate conservation concern and prioritize gaps in penguin research and conservation. Workshop attendees represented 8 countries and a broad range of expertise on penguins (Supporting Information). Recognizing that a lack of consensus among scientists on priorities and approaches can impede conservation (e.g., in the case of African penguin *Spheniscus demersus*: Holcombe 2015), our goal was to foster conservation action on behalf of penguins through intensive discussions and structured ranking processes.

## Priority Species

In a facilitated session, we drew on our collective expertise (Supporting Information), published literature, and insight from collaborators to identify conservation and research needs. We grouped needs into broader themes (e.g., research on microplastics and harmful algal blooms under the marine‐pollution theme) (Supporting Information), which led to 9 conservation and 12 research priorities. These discussions were informed by García Borboroglu and Boersma (2013), Trathan et al. (2015), and a 2016 IUCN SSC PSG workshop (Boersma et al. 2017; IUCN SSC PSG 2017).

We used a modified pairwise‐ranking approach to prioritize the identified needs (e.g., Thurstone 1927; Kendall & Smith 1940; Jones 1995). First, we used the criterion general importance to penguins to conduct pairwise comparisons. For each pair, committee members voted for the need they considered of higher priority. We tallied our votes and calculated weighted scores by dividing the number of votes for each priority by the total number of votes available. As a group, we decided which species each priority applied to (Supporting Information). For the final rankings, we multiplied the weighted scores by the number of relevant species (Table 2). Therefore, the highest ranked threats were those that had the most votes and were considered relevant to all or most penguin species.

**Table 2:** Ranked priorities for penguin research and conservation

	Pairwise ranking score	No. of relevant species	Final weight	Ranking
Research				
population surveys	0.13	18	2.34	1
demographic	0.10	18	1.8	2
environmental patterns	0.09	18	1.62	3
fisheries interactions	0.14	11	1.54	4
foraging ecology	0.08	18	1.44	5
natural history	0.10	13	1.3	6
marine pollution	0.08	11	0.88	7
diet composition	0.05	17	0.85	8
human impacts	0.08	7	0.56	9
interspecific interactions	0.04	7	0.28	10
taxonomy review	0.07	4	0.28	11
disease surveillance	0.03	4	0.12	12
Conservation				
marine spatial planning	0.20	18	3.6	1
species action plans	0.16	16	2.56	2
public awareness	0.11	18	1.98	3
disease management	0.09	17	1.53	4
introduced species	0.14	6	0.84	5
tourism regulation	0.10	8	0.8	6
nesting habitat	0.10	4	0.4	7
natural predators	0.06	3	0.18	8
harvesting or trade	0.04	2	0.08	9

We acknowledged that the species facing the greatest number of conservation and research needs may not be the species of the most immediate conservation concern. Therefore, we also voted on which species were in most pressing need of policy intervention and international collaboration; we used rapid population declines or extremely limited geographic range as our criteria. Three species were unanimously voted as international priorities: African penguin (*S. demersus)*, Galápagos penguin (*Spheniscus mendiculus)*, and Yellow‐eyed penguin (*Megadyptes antipodes)*.

### African Penguins

The global population of African penguins is approximately 21,000 pairs, down from over 1.5 million pairs in the early 1900s (Crawford et al. 2011). This ongoing rapid decline is primarily caused by reduced prey availability (Crawford et al. 2011), attributable to climate change and fisheries (Pichegru et al. 2012; Sherley et al. 2017). Additional threats include petroleum discharge (Fowler et al. 1995; Barham et al. 2007; Wolfaardt et al. 2008), ship‐to‐ship bunkering (South African Department of Environmental Affairs and South African National Parks data), and predation by seals and land‐based predators (Weller et al. 2016; Cape Nature Conservation and South African National Parks data). An ecosystem‐based approach to fisheries management that ensures sufficient prey for African penguins, especially when prey stocks are low, is urgently needed. The recently expanded marine protected area network includes some breeding colonies (Department of Environmental Affairs 2018), but it does not provide the protection necessary for all life stages (Harding 2013; Roberts 2016; Sherley et al. 2017).

### Galápagos Penguins

This rarest of penguin species is restricted to Ecuador's Galápagos Islands. Its population undergoes extreme fluctuations and is of unknown size due to low and variable resighting rates (Boersma et al. 2013). Galápagos penguins do not breed when food is scarce; instead, they spend much of their time foraging at sea. When they do breed, and often when they molt, they are hidden in lava nests where they cannot be seen (Boersma 1978). This population is threatened by severe El Niño events, which are becoming more frequent (Cai et al. 2014), because they increase adult and juvenile mortality and result in breeding failure (Boersma 1978; Boersma 1998; Vargas et al. 2006). When food is abundant, erosion of existing nesting sites, a lack of well‐shaded breeding sites, and invasive predators (e.g., cats and rats) limit successful breeding and population recovery. Removing invasive predators and building predator‐free breeding sites would benefit the breeding population. Given the population's sensitivity to environmental variability, action should be taken to protect the population even in the absence of complete scientific understanding. Improving and enforcing fisheries management is crucial to ensure food availability.

### Yellow‐eyed Penguins

There are approximately 1700 pairs of Yellow‐eyed penguins (Seddon et al. 2013). Populations occur in 2 geographically and genetically distinct management units (<40% on the South Island of New Zealand, >60% on the subantarctic Campbell and Auckland Islands [Boessenkool et al. 2009]). Steep declines are ongoing and projected to continue for mainland populations. Declines are poorly understood but likely driven by introduced predators, disease, environmental change, and fisheries (Alley et al. 2017; Gartrell et al. 2017; Mattern et al. 2017). Subantarctic breeding areas are population strongholds, but basic research on population sizes and trends is lacking, and these populations are threatened by introduced mammals (Challies 1975). Increasing penguin‐focused tourism has increased stress and reduced productivity (e.g., French et al. 2018) and may contribute to disease outbreaks. Of highest priority is developing effective marine spatial planning and tourism planning.

## Conservation and Research Needs for All Penguins

The highest ranked research needs for penguins entail continued population monitoring (estimating demographic rates and population trends) and improved understanding of environmental conditions and change. We also identified research priorities for emerging or growing threats. For example, disease surveillance is increasingly important for several species, particularly for small populations that regularly come into contact with humans through tourism (e.g., spread of zoonotic enteric bacteria [Cerdà‐Cuéllar et al. 2019]). Diseases are a concern for African penguins (Parsons & Vanstreels 2016), Gentoo penguins (*Pygoscelis papua*) (Munro 2007), King penguins (*Aptenodytes patagonicus*) (Cooper et al. 2009), Northern rockhopper penguins (*Eudyptes moseleyi*) (Jaeger et al. 2018), and Yellow‐eyed penguins (Alley et al. 2004, 2017). Other threats likely to be underestimated that require additional research include impacts of bycatch (all penguins [Crawford et al. 2017]), plastic ingestion (e.g., Magellanic penguins [Marques et al. 2018]), and invasive species (all seabirds [Spatz et al. 2017]).

Producing and implementing marine spatial plans (Ehler & Douvere 2009) emerged as the highest ranked conservation priority. Marine spatial planning is a practical approach to ecosystem‐based management (e.g., Lombard et al. 2019) that examines all interactions within an ecosystem, rather than considering single issues, species, or ecosystem services in isolation (Ehler & Douvere 2009). For penguins, this process should identify stakeholders to help map and resolve conflicts and incorporate conventional fisheries management tools, seasonal fisheries closures, and corridors that include migratory routes (e.g., Trathan et al. 2014).

Some conservation needs were restricted to a few species but represent important gaps in knowledge or conservation. For example, penguins at some colonies can face high rates of predation on land (e.g., Little penguins [*Eudyptula minor*] [Colombelli‐Négrel & Tomo 2017]) or at sea (e.g., African penguins [Weller et al. 2016]). Threats to penguins can be manifested in several ways. For example, climate‐associated reductions and shifts in ocean productivity and prey will likely affect all species (Bost et al. 2015; Trathan et al. 2015; Ramírez et al. 2017), but climate change also has region‐ and species‐specific effects. Increasing the intensity and severity of El Niño–Southern Oscillation events affect penguin breeding and body condition (Galápagos penguins [Boersma 1978, 1998]), foraging efficiency and success (Little penguins [Pelletier et al. 2012; Carroll et al. 2016]), and breeding performance (Humboldt penguins [*Spheniscus humboldti*] [Simeone et al. 2002]). High precipitation events cause flooding of burrows (African penguins [Kemper et al. 2007], Humboldt penguins [Simeone et al. 2002], Magellanic penguins [Boersma & Rebstock 2014]), changes in sea ice cover cause range shifts (Adélie penguins [*Pygoscelis adéliae*] [Cimino et al. 2016]), ecological mismatch of juvenile penguins and their prey cause reduced survival rates (African penguins [Sherley et al. 2017]), and ocean temperature anomalies cause mortality during migration (Magellanic penguins [García Borboroglu et al. 2010]).

## Leveraging Science for Penguin Conservation

Of seabird breeding colonies, penguin colonies are among the most intensely researched (e.g., Richdale 1957; Ainley et al. 1983; Crawford et al. 2006; Boersma 2008; Chiaradia et al. 2010; Robertson et al. 2014; Barbraud et al. 2015; Bost et al. 2015). Why, then, has science not always been translated into effective conservation? There are 4 areas for improvement.

First, understanding penguins requires long‐term data sets, but these are rare, usually localized, and often spearheaded by a few individuals working independently. Also, it is difficult to find funding for long‐term studies (Birkhead 2014; Kuebbing et al. 2018). Governmental institutions should strive to maintain long‐term research that goes beyond tracking abundance to include monitoring of ecological processes and other factors key to effective penguin conservation (e.g., as done by the Antarctic Ecosystem Research Division [Trivelpiece et al. 2011; Hinke et al. 2015], Australian Antarctic Division [Emmerson & Southwell 2008; Southwell et al. 2017], and French Polar Institute [Jenouvrier et al. 2014; Bost et al. 2015]).

Second, it is easiest and least expensive to study penguins during the breeding season, when they are central place foragers. For example, 75% of the penguin tracks in Birdlife International's (2018) database occur during the breeding season. However, the nonbreeding season is often marked by higher mortality than the breeding season (e.g., Northern rockhopper and Southern rockhopper [*Eudyptes chrysocome*] [Dehnhard et al. 2013]), and can have carry‐over effects on the breeding season (e.g., African penguins [Sherley et al. 2013], Little penguins [Salton et al. 2015], Magellanic penguins [Rebstock & Boersma 2018], Northern and Southern rockhopper penguins [Thiebot et al. 2012], Macaroni penguins [*Eudyptes chrysolophus*] [Crossin et al. 2010]).

In the nonbreeding season, some species migrate thousands of kilometers, and knowledge of these movements remains limited (e.g., Magellanic penguins [Stokes et al. 2014]; Fiordland penguins [*Eudyptes pachyrhynchus*] [Mattern et al. 2018]) (Table 1). There is especially little knowledge of juvenile life stages because juvenile penguins often prospect at other colonies and remain unobservable at their natal colony for the first few years after fledging or, in some cases, emigrate permanently (e.g., Humboldt penguins [Simeone & Wallace 2014], Magellanic penguins [Stokes et al. 2014]). Improved knowledge of this stage is key to conservation because some species have low juvenile survival rates (e.g., <20% on average for African penguins [Sherley et al. 2018] and Magellanic penguins [Gownaris & Boersma 2019], but >75% for King penguins [Saraux et al. 2011] and Southern Rockhopper penguins [Dehnhard et al. 2014]), which can be a strong driver of population decline (e.g., Magellanic penguins [Gownaris & Boersma 2019]). Penguins in remote regions of Antarctica and the subantarctic or in the sea caves or coastal forests of New Zealand are challenging to study year‐round for all life stages. Technological advances (e.g., satellite imagery) may improve studies of remote colonies (Ancel et al. 2017; Borowicz et al. 2018).

Third, although reproductive success responds more immediately and dramatically to improved resource availability (Oro 2014), population growth rates are most sensitive to changes in adult mortality (e.g., African penguins [Sherley et al. 2018], Magellanic penguins [Gownaris & Boersma 2019]). Thus, adaptive management and protection at broad spatial and temporal scales are required. Most species forage over large areas (e.g., Boersma & Parrish 1999; Bost et al. 2015; Mattern et al. 2017) that vary between the breeding and nonbreeding season (Warwick‐Evans et al. 2018) and sometimes with age class (Sherley et al. 2017). Foraging areas may extend to internationally managed waters and often cross jurisdictional boundaries (e.g., BirdLife International 2018). Safeguarding the future of penguins therefore requires international collaboration on spatial planning, particularly in areas beyond national jurisdiction (Trathan et al. 2018; Warwick‐Evans et al. 2018).

Marine reserves are not a panacea for fisheries management problems. However, when guided by a case‐by‐case understanding of fisheries and ecosystem structure, they can be valuable tools for conservation (Hilborn et al. 2004). Experimental fishing closures surrounding breeding colonies of African penguins, for example, reduce effort by breeding birds during foraging (Pichegru et al. 2010), increase breeding success (Sherley et al. 2015, 2018), and improve chick condition (Sherley et al. 2018). These effects occur despite concerns about the closures, including the displacement of fishing effort (Pichegru et al. 2012), appropriateness of the experimental design (Weller et al. 2014), and spatial (Pichegru et al. 2012) and temporal (Crawford et al. 2013) resolution.

Finally, scientific data are necessary but, in many cases, insufficient to motivate effective conservation (Ropert‐Coudert et al. 2019). Improving the communication of scientific information to decision makers and stakeholders is also required. For example, at a population and habitat viability assessment workshop for Humboldt penguins (Araya et al. 1999), there were highly conflicting points of view between researchers and fisheries managers. Biologists were concerned that being overly optimistic would lead to the decline or extinction of Humboldt penguins, whereas fisheries managers worried that being overly pessimistic would lead to the collapse of fisheries (Araya et al. 1999). Despite this conflict, the workshop was crucial in defining research priorities that would considerably improve the type and quality of data obtained for Humboldt penguins (e.g., Paredes et al. 2003).

In other examples, decades of research on Magellanic penguins (Boersma et al. 2009; Boersma & Rebstock 2009) led to recommendations for the boundaries of a marine reserve (Boersma et al. 2015). However, the science itself did not catalyze conservation action until further efforts were made to engage politicians, legislators, and stakeholders (García Borboroglu et al. 2015). Similarly, the biodiversity management plan for the African penguin is based on a long history of research (e.g., Crawford et al. 2011) and resulted from collaboration among scientists, managers, nongovernmental organizations, and legislators (Department of Environmental Affairs 2013). In the South Indian Ocean, collaboration between scientists and politicians led to the expansion of the marine reserve surrounding the Kerguelen and Crozet archipelagoes (French Decree 2016‐1700). This expansion included the creation of a large no‐take zone (120,000 km^2^) that benefits many marine predators, penguins included. Elsewhere in the South Indian Ocean, tracking of Northern rockhopper and other seabirds supports the recent expansion of a marine reserve now covering the entire Amsterdam Island and St. Paul Island Exclusive Economic Zone (Heerah et al. 2019). These examples show that individual scientists and interdisciplinary species‐specific working groups play important roles as experts on and advocates for the species they study (IUCN 2017). They also highlight that success depends on establishing trust with decision makers.

Penguins occur in most of the Southern Hemisphere's biodiversity hotspots (Ramírez et al. 2017) and act as marine sentinels in these systems (Boersma 2008). The general decline in the population size of many penguin species warns of widespread ecological change across habitats used by penguins and highlights the need for immediate and focused conservation of marine and terrestrial systems alike. Penguins are long‐lived and often disperse widely during the nonbreeding season, characteristics at odds with the current approach to conservation: short‐term funding, small‐scale spatial protection, and lack of effective, internationally coordinated management. Conserving penguins will require creativity, collaboration, and commitment among diverse stakeholders. We, the IUCN SSC PSG, have systematically highlighted and identified research and conservation priorities to move this agenda forward. By fostering communication of and policy action toward these priorities, our goal is to ensure wild penguins exist in perpetuity.

## Supporting information

Details of the affiliations and expertise of the PSG (Appendix S1), descriptions and examples of each conservation and research priority (Appendix S2), and the conservation and research priority needs for the 18 penguin species (Appendix S3) are available online. The authors are solely responsible for the content and functionality of these materials. Queries (other than absence of the material) should be directed to the corresponding author.
