# Supplementary material for: Applying science to pressing conservation needs for penguins
Source: Conserv Biol. 2019 Aug 13;34(1):103–12. doi: 10.1111/cobi.13378 (PMC7027562; doi:10.1111/cobi.13378)
Supplement: Supplementary file 1 — Details of the affiliations and expertise of the PSG (Appendix S1), descriptions and examples of each conservation and research priority (Appendix S2), and the conservation and research priority needs for the 18 penguin species (Appendix S3) are available online. The authors are solely responsible for the content and functionality of these materials. Queries (other than absence of the material) should be directed to the corresponding author. [file COBI-34-103-s001.docx]

**Supporting Information 2**

Descriptions and examples of specific topics fitting under each research and conservation need for penguins, as agreed upon by the Steering Committee of the International Union for Conservation of Nature Species Survival Commission Penguin Specialist Group at a workshop in May 2018.

**Research Needs**

Population surveys – continued research on population sizes and trends with an aim for more consistent methodologies across studies and species

Demographic – estimation of vital rates and how they vary within (e.g. age, sex, breeding state) and across colonies and subpopulations

Environmental Patterns – use of remote sensing and other technologies to track environmental conditions at various spatial and temporal scales and how they are changing over time

Foraging Ecology – better understanding of foraging habitat, both during the breeding and non-breeding season, and how it varies across individuals

Fisheries Interactions – quantification of direct (bycatch) and indirect (habitat modification, competition) effects of fisheries on penguins

Natural History – improved understanding of breeding biology and behavioral ecology of penguins

Marine Pollution – determining the magnitude and impacts of nutrient pollution/harmful algal blooms, microplastics, and petroleum pollution on penguins

Diet Composition – continued use of pre-existing and emerging technologies (stable isotopes, guano DNA, etc.) to monitor the diet of penguins and how it varies across individuals and colonies

Human Impacts – tracking the magnitude and impacts of terrestrial habitat modification and tourism (e.g. penguin stress levels) on penguin breeding sites

Taxonomy Review– further review of significant evolutionary units for penguins (e.g. Should Royal penguins be considered their own species? Should Little penguins be separated into several species?)

Interspecies Interactions – understanding competition with other seabirds for food or nesting sites and quantifying the impacts of predation

Disease Surveillance – continued monitoring of disease outbreaks and their causes and description of new diseases

**Conservation Needs**

Marine Spatial Planning – spatial management including temporal/spatial closures and marine reserves, particularly during the nonbreeding season to reduce negative direct and indirect interactions with fisheries

Species Action Plans – creation of action plans for all 18 species of penguins (currently only available for Northern rockhopper penguins: Project Pinnamin, rzss.org.uk; and African penguins: Biodiversity Management Plan, Molewa 2013) that bring together scientists, NGOs, and governments

Public Awareness – continued outreach with the public and enhanced communication with policy makers

Disaster Management – development of management plans for cases of oil spills, fires, or disease outbreaks

Introduced Species – control and eradication of introduced species on land

Tourism Regulation – management plans for sites where tourists visit (e.g. elevated walkways) and spatial protection of breeding habitats on land

Nesting Habitat – create, restore, and enhance nesting habitat to offset loss of nesting habitat to erosion, climate change (e.g. increased precipitation), deforestation, and other causes of habitat loss

Natural Predators – discussions on how to address natural predators that have returned to areas colonized by penguins when predator numbers were low (primarily cats on land and seals at sea)

Harvesting/Trade – stop trade of penguin species for captive tourist attractions, harvest of penguin eggs, and harvest of penguins as bait in some fisheries
