# Supplementary material for: Applying science to pressing conservation needs for penguins
Source: Conserv Biol. 2019 Aug 13;34(1):103–12. doi: 10.1111/cobi.13378 (PMC7027562; doi:10.1111/cobi.13378)
Supplement: Supplementary file 2 [file COBI-34-103-s002.docx]

**Supporting Information 3**

Key Research and Conservation Priorities for Penguins. Shaded Squares Indicate Presence of a Priority for Each Species. The Three Species in Bold are of the Most Immediate Conservation Concern.

|  | Emperor | King | Adélie | Chinstrap | Gentoo | Macaroni | Royal | N. Rockhopper | S. Rockhopper | Fiordland | Snares | Erect-crested | **African** | **Galápagos** | Humboldt | Magellanic | Little Blue | **Yellow-eyed** |
| --- | --- | --- | --- | --- | --- | --- | --- | --- | --- | --- | --- | --- | --- | --- | --- | --- | --- | --- |
| RESEARCH | | | | | | | | | | | | | | | | | | |
| Population Surveys |  |  |  |  |  |  |  |  |  |  |  |  |  |  |  |  |  |  |
| Demographic |  |  |  |  |  |  |  |  |  |  |  |  |  |  |  |  |  |  |
| Environmental Patterns |  |  |  |  |  |  |  |  |  |  |  |  |  |  |  |  |  |  |
| Foraging Ecology |  |  |  |  |  |  |  |  |  |  |  |  |  |  |  |  |  |  |
| Diet Composition |  |  |  |  |  |  |  |  |  |  |  |  |  |  |  |  |  |  |
| Natural History |  |  |  |  |  |  |  |  |  |  |  |  |  |  |  |  |  |  |
| Fisheries Interactions |  |  |  |  |  |  |  |  |  |  |  |  |  |  |  |  |  |  |
| Marine Pollution |  |  |  |  |  |  |  |  |  |  |  |  |  |  |  |  |  |  |
| Interspecific Interactions |  |  |  |  |  |  |  |  |  |  |  |  |  |  |  |  |  |  |
| Human Impacts |  |  |  |  |  |  |  |  |  |  |  |  |  |  |  |  |  |  |
| Disease Surveillance |  |  |  |  |  |  |  |  |  |  |  |  |  |  |  |  |  |  |
| Taxonomy Review |  |  |  |  |  |  |  |  |  |  |  |  |  |  |  |  |  |  |
| CONSERVATION | | | | | | | | | | | | | | | | | | |
| Marine Spatial Planning |  |  |  |  |  |  |  |  |  |  |  |  |  |  |  |  |  |  |
| Public Awareness |  |  |  |  |  |  |  |  |  |  |  |  |  |  |  |  |  |  |
| Disease Management |  |  |  |  |  |  |  |  |  |  |  |  |  |  |  |  |  |  |
| Species Action Plans |  |  |  |  |  |  |  |  |  |  |  |  |  |  |  |  |  |  |
| Tourism Regulation |  |  |  |  |  |  |  |  |  |  |  |  |  |  |  |  |  |  |
| Introduced Species |  |  |  |  |  |  |  |  |  |  |  |  |  |  |  |  |  |  |
| Nesting Habitat |  |  |  |  |  |  |  |  |  |  |  |  |  |  |  |  |  |  |
| Harvesting/Trade |  |  |  |  |  |  |  |  |  |  |  |  |  |  |  |  |  |  |
| Natural Predators |  |  |  |  |  |  |  |  |  |  |  |  |  |  |  |  |  |  |
